# Convergence of need technology and culture driving the era of physical AI in medicine

**DOI:** 10.3389/fdgth.2026.1914012

**Published:** 2026-09-01

**Authors:** Yonatan Prat, Roee Francos, Moshe Shoham, Eyal Zimlichman, Abraham Tsur

**Affiliations:** 1 Department of Urology, Sheba Medical Center, Tel HaShomer, Ramat Gan, Israel; 2 ARC Innovation Center, Sheba Medical Center, Tel HaShomer, Ramat Gan, Israel; 3 Gray Faculty of Medical & Health Sciences, Tel Aviv University, Tel Aviv-Yafo, Israel; 4 Harvard John A Paulson School of Engineering and Applied Sciences, Cambridge, MA, United States; 5 Faculty of Mechanical Engineering, Technion, Israel Institute of Technology, Haifa, Israel; 6 Dina Recanati School of Medicine, Reichman University, Herzliya, Israel; 7 Gynecology Oncology Department, The Josef Buchmann Gynecology and Maternity Center, Tel HaShomer, Israel

**Keywords:** artificial intelligence, clinical decision making, digital health, human-robot interaction, large language models, physical artificial intelligence, robotics

## Abstract

Medicine is a predominantly physical profession, yet most medical artificial intelligence (AI) remains screen bound. Physical artificial intelligence (PAI) extends AI's capabilities and can directly address many of the healthcare system challenges and unmet needs such as workforce shortages, unsustainable healthcare costs, heightened expectations, aging population and need for pandemic preparedness. PAI relies on systems that autonomously perceive, decide, and actuate in real-time by synthesizing diverse environmental data from multiple sensory sources. These data undergo rapid processing and interpretation, facilitating immediate decision-making and responsive physical actions, including movement, object manipulation, and direct human interaction. This paper first identifies critical healthcare system needs that robotic agents can effectively address. It further examines core actions of PAI systems, emphasizing perceptual pathways, clinical decision-making processes, and human-robot interactions that translate sensory inputs into tailored, patient-specific responses. We explore essential data utilization aspects, including edge-device advances, PAI datasets, digital twins, and edge-to-cloud infrastructures that support real-time inference and are crucial for reducing barriers to PAI implementation. Finally, we analyze the cultural factors accelerating the adoption of PAI in healthcare and review PAI advancements in other sectors. We argue that the convergence of pressing healthcare demands, technological advancements, and cultural readiness signals a tipping point for PAI in medicine.

## 1 Introduction

**Figure 1:**
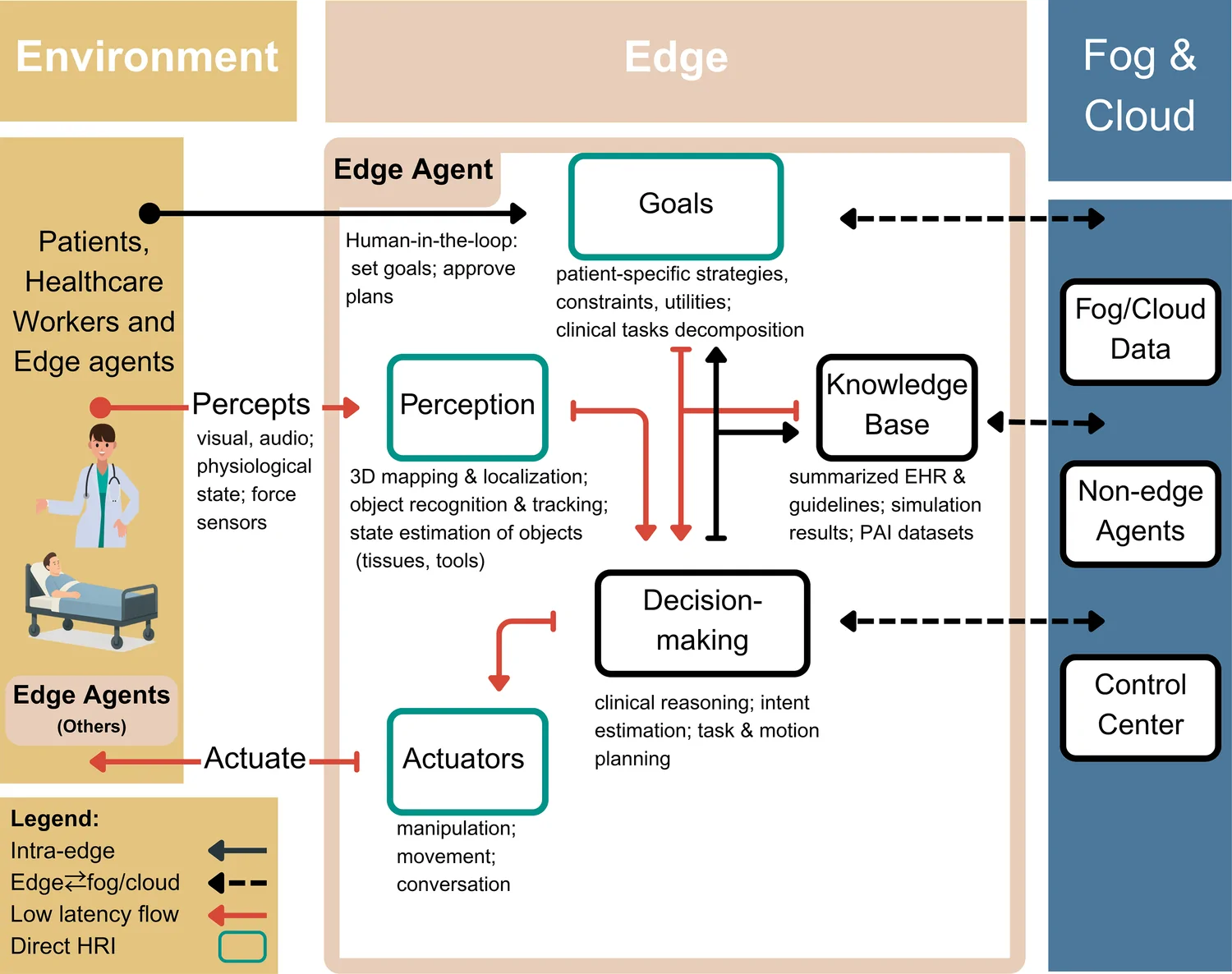
System block diagram of a PAI agent components in clinical settings. **(a)** Overview of the PAI components and action discussed throughout this paper. System block diagram of PAI edge agent executes real-time perception, decision-making, and actuation. It is guided by goals, which can be set by human, and on-device knowledge base (summarized EHR, guidelines, simulation outputs). Fog/cloud services synchronize policies and data, execute heavy workloads (LLMs, digital-twin simulation, safety checks), and coordinate with other agents for longitudinal context and oversight. **(b)** PAI, Physical artificial intelligence; EHR, Electronic Health Record; LLM, Large language models; HRI, Human-robot interaction.

Artificial intelligence (AI) in medicine has developed remarkably over the last decade (1). However, thus far, AI remains predominantly in the digital realm, with limited impact on real-world actions (2). The inherent limitation of this predominance is its inability to translate data-driven insights into direct, physical action. Software-driven AI cannot reposition a patient, adjust a ventilator, disinfect a surface, or perform a suture. In essence, it halts at the gap between knowledge and action. Medicine, however, is intrinsically a physical discipline, in which clinicians frequently perform physical interventions within clinical environments. Physical artificial intelligence (PAI) emerges as a paradigm to extend AI's capabilities by bridging this gap (3). PAI serves as a critical response to pressing system-level urgent needs: an aging global population, chronic workforce shortages, unsustainable healthcare costs, heightened patient expectations, and the imperative of pandemic preparedness. Collectively, these needs underscore the necessity for scalable and adaptive physical solutions that move beyond traditional models of care. PAI agents in medicine (4) autonomously: i) perceive clinical environments using multimodal data from vision, audio, motion, physiologic sensors and electronic health record (EHR); ii) decide by integrating contextual awareness, medical knowledge, and patient-specific information, and iii) interact with patients, clinicians, staff, and other agents through responsive physical actions such as movement, instrument manipulation, or natural conversation. These agents can reside in devices, robots, wearables or smart beds. Firstly, this paper identifies critical healthcare system needs that PAI agents can effectively address. It further examines recent developments that have set the scene for PAI agents through their core actions (5). It emphasizes lightweight perceptual models, clinical decision-making (CDM) processes, and human–robot interaction (HRI) that translate sensory inputs into tailored, patient-specific responses (Figure 1) (6). We explore data infrastructure aspects essential for PAI, edge-devices, PAI datasets, digital twins, and edge–fog–cloud architectures. This infrastructure supports real-time actuation and is crucial for reducing barriers to PAI implementation (7, 8). Finally, this paper appraises cultural and organizational enablers of PAI adoption, including acceptance of transformation, cross-industry advances, institutional strategies, and workforce readiness.

## 2 Challenges and unmet needs

### 2.1 Workforce shortage and burnout

The healthcare industry continues to face significant challenges due to chronic staff shortages and clinician burnout. Recruiting and retaining clinical staff have been identified as primary concerns among healthcare leaders (9). The World Health Organization projects a global shortage of 12.9 million healthcare workers by 2030, including deficits of 4.5 million nurses and 1.94 million doctors (10). This staffing shortfall severely impacts timely access to care, particularly in rural and developing countries (9).‏ PAI has the potential to bridge these gaps by augmenting capabilities and workloads of healthcare providers. PAI agents can automate routine tasks, perform physical examinations with integrated sensors, wound dressing, patient positioning, handle biohazards and do transport tasks. Thus, offloading task from caregivers and reducing patients’ reliance on direct healthcare personnel interventions (11). These functions alleviate routine burdens, enabling medical professionals to focus on critical care activities. Collectively, PAI not only reduces workloads and mitigates burnout but also supports the mental and physical well-being of healthcare staff (1). Ultimately, staff well-being enhanced safety, quality of care, and improved clinical outcomes (12, 13).

### 2.2 Rising costs of healthcare

The current trajectory of healthcare spending poses a significant threat to the sustainability of health systems. In the United States, for example, national health expenditures are projected to grow at 5.6% annually through 2032, outpacing the 4.3% growth in gross domestic product and consuming a projected 19.7% of the 2032 economy (vs. 17.3% in 2022) (14). PAI provides cost-efficient approaches to moderate healthcare expenditures by minimizing operational inefficiencies, automating tasks, expanding access to care, and avoiding secondary costs associated with limited access to care. For instance, sonographer-free robotic systems can perform ultrasound imaging by applying reinforcement learning (RL) to optimize probe trajectory and contact pressure, and by leveraging Large language models (LLMs) to transform the acquired images into automated, structured diagnostic reports (15–18). This system lowers labor costs, makes medical ultrasound more consistent, and expands access to ultrasound modalities in underserved communities. Beyond clinical applications, PAI-driven logistics is poised to minimize operational inefficiencies and resource waste, by automating material transport, inventory management, and sterile supply chains. This momentum is amplified by logistics-intensive sectors. Through scalable, data-driven optimizations, PAI can lowers labor expenses and operational overhead. It offers a promising path to improve economic efficiency and relieve financial pressures on healthcare systems.

### 2.3 Patient expectations

Patient expectations in healthcare are steadily rising, with individuals seeking personalized, responsive, and seamlessly technology-enabled care that enhances outcomes. Surveys show that patients value tools that support self-management, real-time feedback, meaningful engagement, and communication that culminate in a more positive emotional experience (1, 19, 20). These expectations align closely with emerging PAI capabilities (1). PAI platforms (e.g., robots, wearables, and bedside devices) can surface early deterioration, initiate alerts to care teams, guide tailored therapy, and automate elements of chronic-disease management (21–23). They are being used not only to monitor but also to deliver therapies and improve function (23, 24). For example, insulin delivery trials highlight PAI-enabled systems that integrate sensing, decision-making, and physical insulin delivery (21, 22, 25). Additionally, PAI can be used with existing remote-care interventions that have been linked to better adherence, safety, and reduced utilization during transitions from hospital to home (26, 27). Beyond monitoring, socially assistive robots address feelings of loneliness and sustain engagement in lifestyle interventions (28–30). For instance, a small pilot randomized clinical trial found that a humanoid robot could deliver nutrition-counseling sessions to adults with obesity and achieve weight-loss outcomes comparable to clinician-led sessions (30). Moreover, robot-assisted surgery systems (RAS) are expanding across specialties. Large cohort analyses show rapid diffusion of robotic approaches, and comparative studies in selected procedures suggest high patient satisfaction, and reduced morbidity or length of stay (31, 32).

### 2.4 Aging population

The aging global population presents one of the most pressing healthcare challenges of the 21st century. By 2050, the percentage of individuals aged 65 and over is projected to increase by 72%, from 9.3% in 2020 to approximately 16% (33). This demographic shift is closely associated with a growing prevalence of chronic diseases, including diabetes, cardiovascular diseases, and dementia. These conditions necessitate sustained and resource-intensive medical management. Consequently, this trend intensifies existing healthcare pressures by significantly raising demand for services amid persistent staffing shortages. PAI therefore emerges as a transformative solution capable of addressing the complex needs of aging populations. For example, through physical engagement, humanoid agents can enhance cognitive and emotional well-being by mitigating isolation and mental health challenges (28, 29). They can detect disease progression, support nutrition and medication management, facilitate remote care, and ultimately lessen the burden on healthcare systems (26, 30).

### 2.5 Pandemic preparedness

The COVID-19 pandemic drastically reshaped healthcare, exposing numerous needs and accelerating the adoption of digital health solutions (9, 34). The crisis highlighted gaps in healthcare infrastructure, such as inadequate access to care, overwhelmed hospital systems, and shortages of critical resources like personal protective equipment and ventilators (35). These challenges emphasized the need for more resilient, adaptable healthcare systems. The pandemic accelerated the deployment of autonomous ultraviolet robots for facility disinfection and robotic systems for contactless delivery of supplies, minimizing human contact to mitigate viral transmission. Among isolated elderly populations, social robots have been shown to improve medication adherence, encourage physical activity, and facilitate social interactions, thereby enhancing well-being and reducing the burden on healthcare systems (28, 36). PAI deployed in these contexts protects healthcare workers from infection risks and ensures continuity of care when human resources are strained.

## 3 Bridging AI and the physical world

### 3.1 AI frameworks-Jobs-to-be-done

#### 3.1.1 Perceiving

A critical foundation for PAI in healthcare is the ability of PAI agents to perceive, in real time, a variety of signals - visual, auditory, tactile, infrared, and ultrasound - via reliable sensors. These data are then processed by AI models, primarily from the field of computer vision (CV) (1). CV often requires powerful deep neural networks (DNNs), especially to process large volumes of data accurately, for example, hundreds of ultrasound images to determine the optimal probe position (18). DNN architectures endow PAI agents with the ability to classify what they “see,” “hear,” and “sense”. Standard DNN architectures, such as convolutional neural networks (CNNs) and vision transformers (ViT), have shown impressive performance in tasks like image recognition, medical imaging analysis, and speech recognition (17, 27, 37). However, DNNs often require substantial computational power, introducing latency that can disrupt clinical workflows or affect patient safety. Recent innovations in lightweight DNN models aim to reduce the number of parameters while maintaining high accuracy in tasks such as ultrasound guidance or surgical tool identification (23, 32, 38–40). Back in 2019, state-of-the-art CNN models commonly exceeded 50–100 million parameters, driving up computational load and latency. Today's efficiency-oriented models reach comparable or better accuracy with far fewer parameters. For instance, a Stanford-led study on 3D understanding shows that a compact model with just 1.4 million parameters hits 91.5% accuracy on a leading real-world benchmark, raising the bar for lightweight architectures (41). As an example, surgical-phase recognition, patient monitoring, and instrument tracking can be performed at near-instant speeds (39). Although this demonstration supports the technical feasibility of lightweight perception architectures, it does not constitute evidence of clinical effectiveness or safety (23, 42). Clinical translation will require prospective validation under representative healthcare conditions**.** The integration of lightweight models with specialized edge hardware enables PAI agents to achieve their goal by dynamically gathering, processing, and interpreting environmental multimodal data continuously within sub-second intervals. This ensures that sensory inputs directly contribute to informed and real-time CDM, allowing immediate clinical feedback.

#### 3.1.2 Deciding

Physical embodiment and automation alone do not necessarily constitute PAI (3). Conventional robotic systems primarily translate human commands into precise physical actions, whereas AI-assisted systems use computational models to provide guidance, predictions, or limited task execution while clinicians retain responsibility for planning and approval (3, 4). Autonomous embodied PAI agents represent a higher level of integration, combining perception, patient-specific decision-making, physical action, and real-time adaptation within defined clinical constraints (6). The autonomy of AI systems in healthcare is often classified into levels, a framework which (in the context of PAI) can be illustrated by RAS (8, 43, 44). Most FDA-cleared RAS still sit at Level 1 autonomy: the surgeon must generate, select, execute, and continuously monitor every maneuver (6, 45). In practice, these systems function as highly precise “smart instruments” providing tremor filtration, tool tracking, or motion constraints, like no-fly zones, yet offering no patient-specific strategies (1, 6, 31). A limited subset has progressed to Level 2 task autonomy, where robots provide guidance based on pre-operative planning or run pre-programmed tasks, such as knot-tying or bone drilling, once the surgeon supplies all parameters. Yet, they remain incapable of planning or adapting on their own. True adaptation emerges only at Level 3 conditional autonomy, achieved by roughly 6% of cleared platforms (6). At this stage, the RASs mine pre-operative imaging, suggest several tailored plans, execute the one the surgeon approves, and updating it intra-operatively without repeated prompts. No commercial system has advanced to Level 4 or Level 5 (6). In these levels robots would independently set goals and constraints, then, generate, choose, and change operational strategies in real-time, based on EHR and ongoing operational state. It should be mentioned that crossing higher levels of autonomy is largely dependent on the regulation route, as surgical robot companies are pushing for higher autonomy.

RL drives agents to select actions in a dynamic environment to maximize expected cumulative reward (40). RL comprises of a series of actions that help agents achieve their goals (4). RL and Natural Language Processing (NLP) models push PAI systems toward higher levels of autonomy by coupling control strategies with patient-specific reasoning and CDM (6, 17, 22, 42, 46–49). First, RL-NLP were trained with LLMs on spoken/written, such as instructions and via digital-twin simulations. They generate control strategies that transfer to real-time clinical settings actuation (32, 48, 49). Additionally, they directly address setbacks typical for PAI agents in medical environments, where safety, low-latency, efficiency, data privacy and adaptability to infrastructure are crucial (7). These control strategies may allow RAS to adapt to unseen cases, like patient anatomic variability or unanticipated changes during surgery, by drawing information from EHR or verbal instructions by clinicians (4, 5, 42). As a second cornerstone, advances in CDM enable robots to synthesize patient-specific sequential decision strategies (5, 50). For example, a study showed a LLM (ChatGPT-o1 preview) demonstrated superior performance to physicians on care-planning benchmarks (50). As a further illustration, in August 2025, a benchmark was established when a clinical-decision support platform achieved a 100% score on of the United States Medical Licensing Examination on non-pictorial items (51). These results represent performance on controlled cognitive benchmarks rather than prospective clinical evidence, and they do not demonstrate that an LLM can safely direct robotic actions or independently manage patients. However, this capacity of CDM, demonstrated in cognitive tasks, is the critical prerequisite for robotic systems to independently formulate and adapt patient-specific plans (4). RL and multimodal LLMs allows PAI to generate tailored strategies based on data derived from multiple channels and agents, leveraging clinician-level reasoning to enhance the level of autonomy needed for PAI agents to impact healthcare (5, 52). Lastly, to enable real-time inference, PAI agents will depend on an edge-resident, updatable knowledge base that integrates summarized EHR data and clinical guidelines, simulation outputs, and PAI datasets (4, 39, 53, 54).

#### 3.1.3 Interacting

Teamwork in clinical practice depends on interaction that spans explicit verbal instructions, nuanced contextual cues to physical gestures. Likewise, PAI systems require robust HRI to enable safe, efficient collaboration between robotic agents and healthcare professionals (7, 15, 48, 49, 52). HRI frameworks equip robotic systems to keep human-in-the-loop: clinicians can set goals, approve plans, and take over control by interpreting spoken commands, and receive workflow updates (49). Beyond overt interactions, DNNs allow robots to decode subtler signals, often critical to real-world actuation, such as hand gestures, posture, and gaze direction, thus supporting adaptive, context-sensitive collaboration at the point of care (3, 25, 37, 48, 55). Established usability methodologies, including early identification of end users, workflow-based testing, can guide iterative evaluation and deployment of these systems (56–58). In rehabilitation, for example, robots can dynamically tailor exercise protocols by integrating covert pain cues with patients’ verbal feedback. The ability to parse subtle signals is also critical for user acceptance, as it shapes the perceived “personality” of PAI agents among patients and staff (19). Once inputs are interpreted, RL algorithms determine how tasks are executed safely and effectively, while policy updates allow robots to anticipate recurrent requests or individual preferences (32, 46). NLP further extends HRI by lowering interaction barriers: small language models (SLMs) and edge-deployed LLMs enable near-patient deployment with low latency and facilitate multi-agent workflows (5, 53, 59–62). Clinicians might request, “recall the patient's last hemoglobin reading,” or a surgeon could instruct a RAS to “elevate arm #2 by 3 cm” (57, 63). In response, LLM-powered robots can provide context-rich insights, such as highlighting potential risks based on recent imaging (4, 31). Moreover, emerging approaches such as retrieval-augmented generation (RAG) and LLM-to-SLM agent conversion further strengthen collaborative decision-making (4, 5, 15, 53). In unpredictable scenarios, for instance, a rare intraoperative complication, a PAI agent may use a lightweight SLM to communicate with non-edge agents, accessing medical guidelines, updating its knowledge base, and offloading compute-intensive tasks (53). Physical AI extends beyond software-based reasoning or collaboration among LLM agents by integrating perception, decision-making, and physical action within an embodied, real-time system capable of interacting with patients, clinicians, and the healthcare environment (62).

### 3.2 The role of data

Robust, adaptive PAI in healthcare depends on high-quality, diverse, reliable, and accessible data that reflects real-world clinical and physical complexities and can be processed in real-time (3). In that context, four aspects stand out:

#### 3.2.1 Edge devices advancement

Algorithmic breakthroughs dominate headlines, yet PAI hinges just as strongly on steady, often overlooked hardware progress. This progress does not simply follow Moore's Law; it represents architectural advancements that enable robots and AI models to function effectively at the point of care on edge-processors (39, 64, 65). Initially, general-purpose graphics processing units (GPUs) unlocked modern AI by speeding the matrix multiplications central to DNN training and inference (39). Today, AI-specific GPU and dedicated accelerators, like Tensor Processing Units (TPUs) and application-specific integrated circuits (ASICs), deliver faster, lower-power inference, shrinking the cost and footprint of robotic platform (65). For reference, in early 2025, a $250 investment in edge-AI computer delivers 67 Tera Operations per Second (TOPS) of performance, 102 Giga Bites per Second (GB/s) memory bandwidth, and power consumption of 15 Watts (Table 1). In 2015, for comparison, the similar outlay yielded only 1 TOPS and 25.6 GB/s, with the same power consumption. TOPS and TOPS-per-watt directly determine inference latency and efficiency, critical factors for DNNs (CNNs, ViTs and LLMs) pipelines which play a key role in PAI. High throughput per watt also allows a full clinical shift to be powered by a compact, fan-less battery, eliminating trailing cables, while sealed housing meet infection-control standards and cut maintenance.

**Table 1:** 2025 vs. 2015 performance comparison.

Parameter	Jetson Orin Nano Super Developer Kit (2025)	NVIDIA Tegra X1 (2015)
Cost	$250	∼$300
Performance	67 TOPS	∼1 TOPS
Memory bandwidth	102 GB/s	25.6 GB/s
TDP	∼15 W	∼15 W

Performance comparison of processors 10 years ago and now.

TOPS, Tera operations per second; GB/s, giga bites per second; TDP, thermal design power; W, watt.

#### 3.2.2 PAI dataset

To manipulate or navigate physical objects safely, a robot needs more than just static images or text-based instructions. It requires multimodal information of 3D spatial relationships, accurate self-localization, force feedback, tissue interactions, and object properties, named PAD (41, 54, 66, 67). PAD comes from real-world demonstrations, for example, surgeons using laparoscopic instruments or polyp removal in colonoscopy, or from simulations (also known as digital twins) that meticulously mirror real scenarios (32). Imitation learning uses such data to train models that replicate expert behavior. Whether it is suturing in minimal-access surgery or positioning a syringe, properly annotated demonstrations can significantly cut down the time robots need to reach expert-level performance (45). However The central challenge is no longer whether machines can represent the world, but whether they can act within it with robustness, flexibility and purpose. Meeting that challenge will require integrating insights across disciplines that have often developed separately (3).

#### 3.2.3 Digital twins and synthetic data

A digital twin is a reactive virtual replica of a physical asset, such as a robot, or a patient, an operating room scenario, and even an entire hospital ward. Digital twins allow clinicians and researchers to reproduce clinical events at scale. This scaling improves model training and enhance decision-making capabilities, all without putting real patients at risk (3, 67–70). For example, during colonoscopy, polyps of diverse shapes and positions can be introduced into the digital-twin simulation, prompting a PAI agent to adapt its detection and resection strategies (32) These agents not only learn from existing PADs but also exploit digital-twin environments to model complex interactions, such as rigid-instrument contact with tissue, to generate more PAD (66). Moreover, synthetic data augmentation is poised to alleviate data scarcity and imbalances. These setbacks often limit deployment in rare diseases, low-income settings, and infrequent scenario, thereby advancing equity and expanding treatment options (70, 71). A notable digital-twin modeling environment is Genesis, an open-source platform created by researchers at several collaborating institutions (72). It fuses PADs, high-fidelity digital-twin representations, and LLM-powered reasoning to accelerate physical simulation of PAI agents. This platform packages complex simulation, control, and analytics tools behind a low-code interface. It sharply reduces technical barriers for researchers, developers, and clinicians looking to explore and iterate on PAI technologies (73).

#### 3.2.4 Data transmission and computing: edge, fog, and cloud

**Figure 2:**
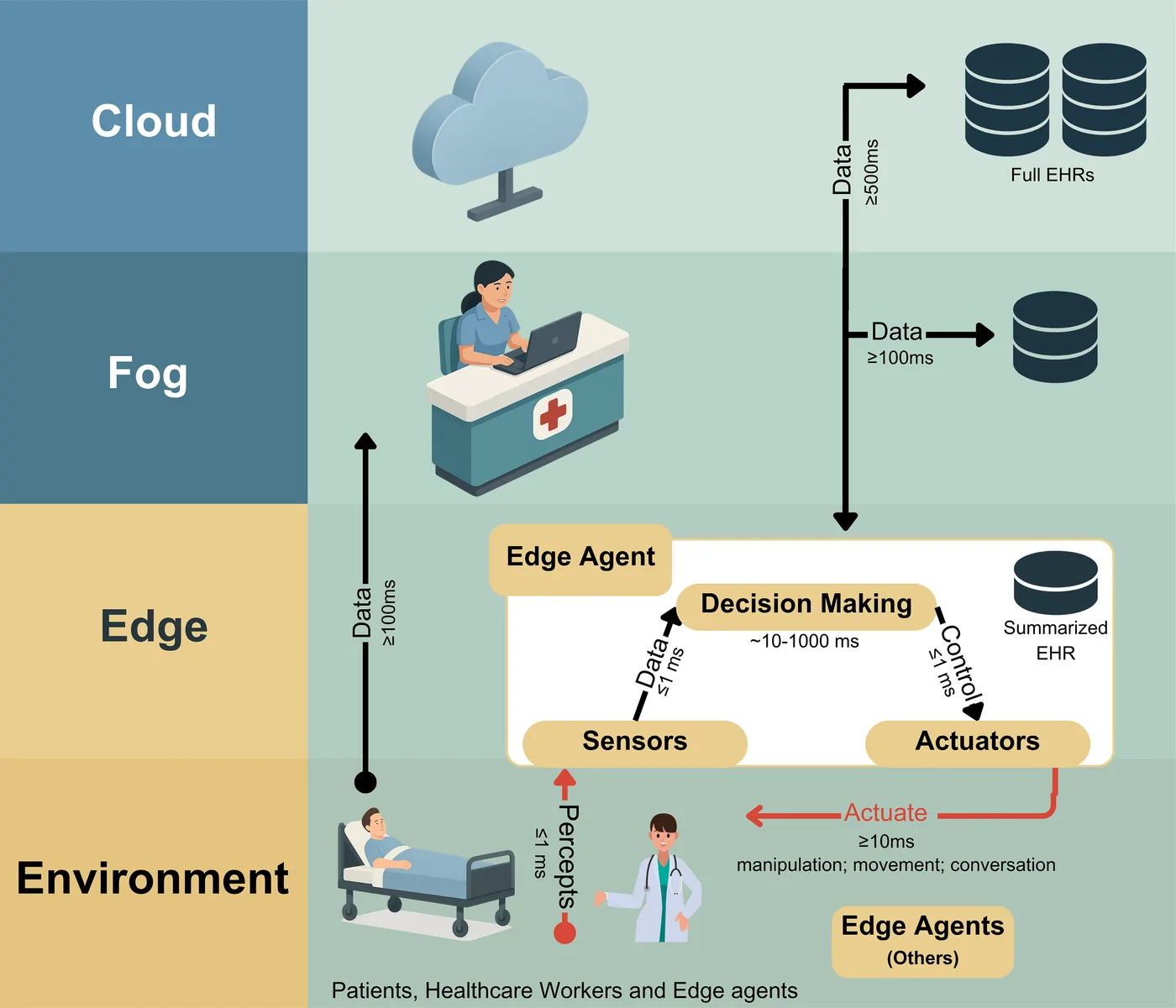
A three-tier edge-fog-cloud architecture in healthcare. The edge layer facilitates real-time decision-making an actuation, supported by fog layer and orchestrated by cloud layer. The actuation pathway shows the direct interaction between the system and the individuals in the environment. Ms, Millisecond (typical time to complete); EHR, Electronic Health Record. 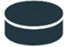
- Icon size is proportional to the available computational power and storage.

**Figure 3:**
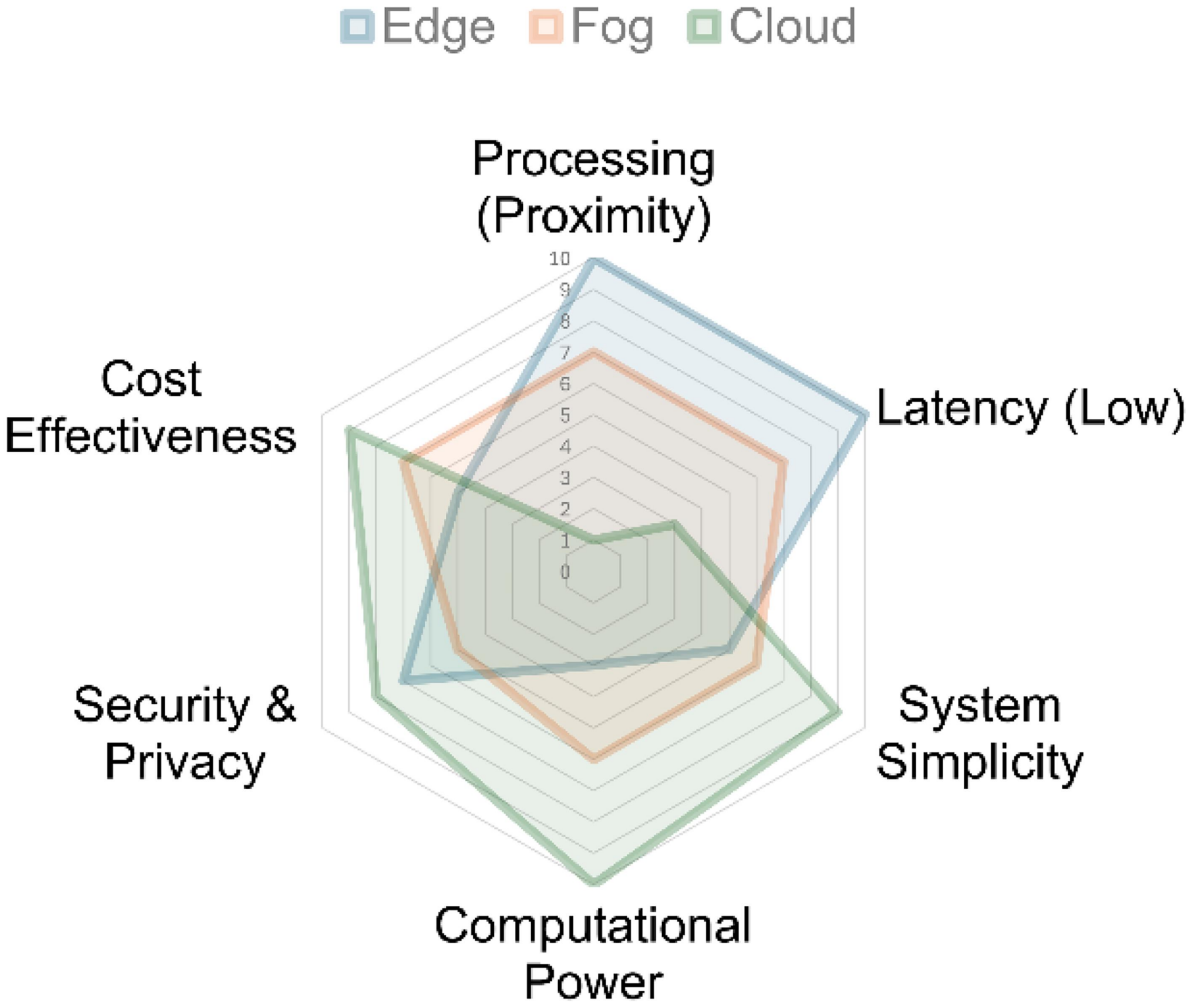
Ranking of edge, fog, and cloud by key parameters.

Applying PAI in healthcare relies on a tiered data pathway that keeps decisions close to the bedside while harnessing large-scale analytics in the background (Figure 2). It demands a balance between computational speed, data security, and scalability (7, 27). Edge computing, fog computing, and cloud computing each fulfill a vital yet distinct role in delivering seamless PAI solutions. These paradigms differ primarily in terms of (a) location of data processing, (b) latency, (c) computational power, (d) security and privacy, (e) system complexity, and (f) cost effectiveness (Figure 3 and Table 2) (7). Edge layer locates data processing directly within the PAI agents; humanoid robot, RAS, bedside monitors, wearables, and ventilator machine. Edge nodes support light-computation tasks, storing relevant PAD and summarized patient data temporarily. They deliver insights on-site, which is vital for rapid decision-making and latency-sensitive healthcare applications (7). Edge solutions provide near-instant feedback for interventions such as RAS (39), or harnessing NLP tools for HRI (53, 60). For instance, in robotic surgery, edge nodes have demonstrated sub-second surgical-phase recognition using lightweight CV, which potentially improve surgical CDM and reduce operative risks (39). Edge nodes are beneficial for Internet of Things (IoT) integrations, where connected sensors generate patient data (27). Sitting one step upstream, fog layer serves as an intermediary layer, it resides near the originating devices, and bridges centralized cloud resources and local edge devices. By putting computational resources closer to data origins, fog layer optimizes latency-sensitive tasks by minimizing unnecessary data transfers to cloud. For example, when deployed on fog networks, raw patient data (e.g., EHR) are transformed into clinically meaningful insights, interpretable by clinicians and agents (4, 57, 59). Subsequently, these insights can be shared with PAI agents operating at the edge nodes. Further upstream, the cloud layer offers vast computational power and storage capacity. However, its inherent latency poses challenges for time-critical clinical applications and necessitates data protection protocols (7). The cloud layer is therefore well-suited for two key functions: first, handling data-intensive, latency-tolerant tasks such as digital-twin simulation, medical image computing and remote monitoring; and second, performing data orchestration between cloud, fog and edge layers. This orchestration involves pre-processing, synchronization, and distribution of data between layers, such as de-identified EHR summarizations, patient-specific strategies and PAD (4).

**Table 2:** Ranking of edge, fog, and cloud by key parameters.

Parameter	Edge	Fog	Cloud
Processing (proximity)	10	7	1
Latency (low)	10	7	3
System simplicity	5	6	9
Computational power	3	6	10
Security & privacy	7	5	8
Cost effectiveness	5	7	9

## 4 Factors driving a cultural shift

### 4.1 Fast-tracking new paradigms

Healthcare is undergoing a rapid transformation, embracing new clinical and operational paradigms. Both caregivers and patients are increasingly open to reconsidering long-standing clinical traditions. The growing adoption of remote care and software-based AI exemplifies cultural shifts that once seemed improbable (26, 30). A similar trajectory can be expected with PAI. Patients increasingly recognize and prefer robotic-assisted interventions, reflecting confidence in the precision, reproducibility, and safety of robotic systems (19). This demonstrates a broader societal readiness to normalize robotic autonomy within clinical practice. Although concerns persist regarding the expanding role of robotics and automation in healthcare, accumulating clinical evidence is likely to highlight demonstrable patient-centric outcomes, including improved access to care, enhanced efficiency, and the advancement of personalized medicine.

Nevertheless, cultural acceptance must be accompanied by regulatory pathways that reflect the distinctive risks of systems capable of physical action (44). Adaptive PAI systems that undergo software updates or continue learning after deployment may challenge conventional approval processes because their performance and behavior can change over time. A total-product-lifecycle approach should therefore include predetermined change-control protocols, version traceability, revalidation after material modifications, and continuous post-market monitoring for performance drift, adverse events, and subgroup-specific failure (73). Ethical governance must evolve in parallel. Patients should be informed when autonomous or semi-autonomous systems participate in their care, while governance frameworks should define requirements for meaningful human oversight, transparency, auditability, and protection of patient autonomy and dignity (44) Clear allocation of accountability among clinicians, healthcare institutions, manufacturers, and software providers, supported by multidisciplinary ethics and safety committees, may reduce uncertainty and strengthen public confidence in PAI adoption.

### 4.2 Global shifts outside health systems

The momentum of PAI in healthcare is markedly influenced by the maturation of PAI in other sectors. In logistics and advanced manufacturing, for example, autonomous robots are already essential components of efficient, resilient operations. These industries offer proven use cases in HRI and dynamic navigation, creating a rich playbook of best practices (48, 54, 72). Significant global research and investments, most notably in China, are accelerating this trajectory, driving PAI forward and reducing costs across industries. Morgan Stanley forecasts that, by 2050, the global humanoid robotics market could reach approximately US$5 trillion, with up to 930 million units deployed, primarily for repetitive task execution (74). This technological and economic momentum from the broader landscape is a direct catalyst, paving the way for a future where PAI is a trusted and integral component of patient care. However, healthcare cannot directly adopt industrial systems without adapting them to stricter clinical safety requirements. Connected robotic platforms may be vulnerable to unauthorized access, ransomware, sensor spoofing, denial-of-service attacks, model manipulation, or compromised software updates, any of which could disrupt care or alter physical behavior (75). Resilience should therefore be established through security-by-design, encrypted communication, network segmentation, authenticated updates, continuous threat monitoring, incident-response protocols, and predefined safe-shutdown states (75). PAI systems may also fail through errors in perception, reasoning, communication, navigation, or mechanical actuation, particularly when operating under unfamiliar or out-of-distribution conditions (8, 44). Safety engineering should include structured hazard analysis, redundancy of safety-critical functions, uncertainty and anomaly detection, simulation-based stress testing, physical constraints, manual override, and systematic investigation of adverse events and near misses (44, 61). Lessons from aviation, manufacturing, and other safety-critical industries may provide useful frameworks, but these approaches must be prospectively validated in clinical environments. Large-scale robotic AI may additionally carry environmental costs through model training and inference, cloud connectivity, data storage, cooling, hardware production, battery use, and electronic waste (8, 61). These effects may be mitigated through energy-efficient and task-specific models, appropriate edge processing, renewable-powered infrastructure, durable and repairable hardware, and transparent lifecycle reporting of energy consumption, emissions, and material use (8, 61).

### 4.3 Strategic evolution and institutional readiness

A key driver for this cultural shift is strategic foresight within healthcare institutions. Organizations are moving beyond reactive technology adoption to proactively building comprehensive AI strategies that address clinical efficiency, workforce support, and operational excellence (76). These existing frameworks, including established leadership commitment and multidisciplinary AI committees, provide an ideal platform to expand the strategic vision to include PAI. The ongoing digital transformation has already initiated the necessary conversations around data governance, ethics, and workflow integration. The introduction of PAI in medicine represents a logical and powerful extension of this work, moving from optimizing digital processes to enhancing the physical involvement in intrinsically physical field. By leveraging their current strategic planning momentum, institutions can thoughtfully address the unique requirements of PAI, such as spatial logistics and HRI protocols, ensuring a cohesive and well-managed transition.

Institutional readiness must include explicit consideration of health equity. High acquisition and maintenance costs, limited connectivity, insufficient technical support, and models developed using poorly representative datasets may restrict access or reduce performance in low-resource settings (70). Without deliberate planning, PAI could widen existing disparities by concentrating advanced capabilities in well-resourced centers. Local validation, infrastructure-appropriate systems, inclusive procurement, workforce capacity-building, shared regional platforms, and partnerships with low-resource institutions may support more equitable deployment (70). Economic sustainability is similarly important. Cost-effectiveness and budget-impact analyses should account not only for acquisition costs but also for integration, training, maintenance, cybersecurity, downtime, infrastructure upgrades, and the consequences of system errors (8). Clear reimbursement pathways and value-based payment models should reward demonstrated improvements in outcomes, access, efficiency, or workforce capacity rather than the use of robotic technology alone. Pilot implementation with predefined clinical and economic endpoints may help institutions determine whether broader deployment is justified.

### 4.4 AI-competent workforce

The widespread implementation of AI has underscored the importance of continuous workforce education, a principle that might be vital for the adoption of PAI (2). Current training initiatives, which enhance digital literacy and build confidence in AI-assisted decision-making, are creating a workforce that is increasingly adaptable and receptive to technological partnership. This represents the first stage of a broader evolution in clinical competencies. The next stage, we believe, will build upon this foundation, expanding training to include the skills needed for effective HRI such as collaborating with autonomous systems and managing their operation in dynamic clinical settings. By integrating PAI-focused modules into ongoing professional development and medical and nursing curricula, healthcare can cultivate a future workforce that is prepared to leverage PAI tools to augment their capabilities and enrich patient care.

Clinician acceptance will depend not only on technical performance but also on usability, workflow compatibility, preservation of professional autonomy, and confidence that PAI supports rather than disrupts patient care. Poorly integrated systems may increase workload, create alarm fatigue, or promote either excessive reliance on automation or inappropriate distrust. End-user co-design, usability testing, simulation-based training, competency assessment, staged implementation, and accessible technical support can improve workflow alignment and promote appropriately calibrated trust (8). Integrating these competencies into professional development and medical and nursing curricula may prepare clinicians to collaborate safely with PAI while preserving meaningful human oversight and the interpersonal dimensions of care.

## 5 Summary and conclusions

**Figure 4:**
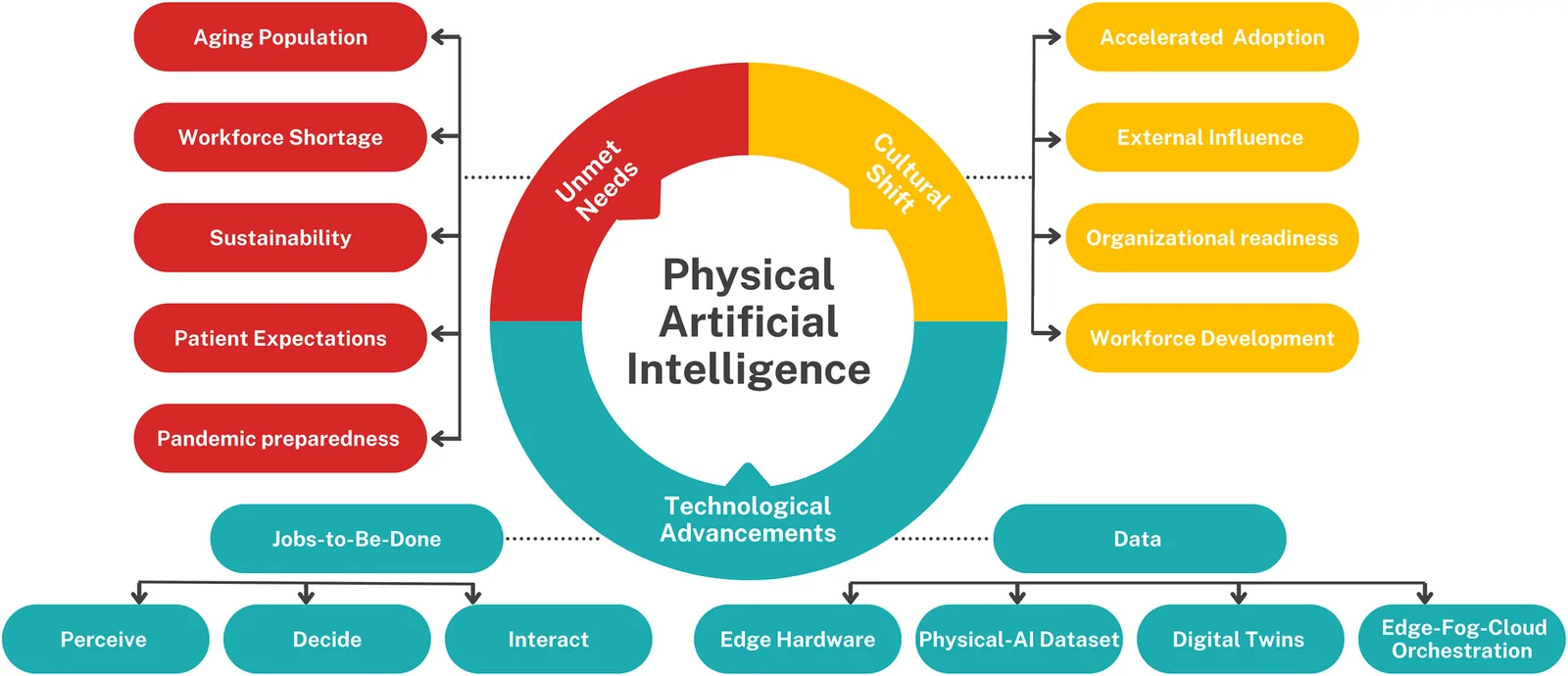
The 3 drivers of physical AI: unmet needs, technological breakthroughs, and cultural.

Healthcare systems are becoming increasingly prepared to adopt PAI, driven by the convergence of mounting healthcare pressures, enabling technological advances, and growing cultural readiness. Collectively, these forces indicate that healthcare may be approaching a critical inflection point in the integration of PAI (3). PAI offers a system-level solution: integration of real-time multisensory perception, reasoning abilities, and HRI to guide appropriate clinical actuation (4). Technological progress has moved PAI closer to patients and caregivers. Compact models execute on edge hardware for perception, decision-making, and interaction; coordinated edge–fog–cloud pipelines deliver NLP, reasoning, and digital-twin simulations trained on PAD; together they drive precise, patient-specific actuation. Culture has caught up: health systems now fast-track new paradigms, PAI developments in adjacent domains are shaping institutional strategies, and both professional training and public perception are shifting toward technology-enabled care. Collectively, these forces are accelerating the transition toward broader adoption of PAI in healthcare (Figure 4). We believe that the next step for health systems will be to adopt a staged approach to PAI integration:(i) systematically evaluate safety, clinical effectiveness, economic value, and scalability across validated use cases; (ii) invest in the requisite data infrastructure, edge–fog computing capabilities, and robust model-lifecycle governance; and (iii) align AI-literacy initiatives and workforce development with the specific collaborative roles envisioned for PAI agents.

## Data Availability

The original contributions presented in the study are included in the article/Supplementary Material, further inquiries can be directed to the corresponding author.
